# Activation of Most Toll-Like Receptors in Whole Human Blood Attenuates Platelet Deposition on Collagen under Flow

**DOI:** 10.1155/2023/1884439

**Published:** 2023-01-17

**Authors:** Y. Liu, S. L. Diamond

**Affiliations:** Department of Chemical and Biomolecular Engineering, Institute for Medicine and Engineering, University of Pennsylvania, Philadelphia, PA 19104, USA

## Abstract

Platelets have toll-like receptors (TLRs); however, their function in thrombosis or hemostasis under flow conditions is not fully known. Thrombin-inhibited anticoagulated whole blood was treated with various TLR agonists and then perfused over fibrillar collagen using microfluidic assay at venous wall shear rate (100 s^−1^). Platelet deposition was imaged with fluorescent anti-CD61. For perfusion of whole blood without TLR agonist addition, platelets rapidly accumulated on collagen and eventually occluded the microchannels. Interestingly, most of the tested TLR agonists (Pam_3_CKS_4_, MALP-2, polyinosinic-polycytidylic acid HMW, imiquimod, and CpG oligodeoxynucleotides) strongly reduced platelet deposition on collagen, while only the TLR4 agonist endotoxin lipopolysaccharide (LPS) enhanced deposition. Following 90 sec of deposition under flow of untreated blood, the addition of various TLR-7 agonists (imiquimod, vesatolimod, and GSK2245035) all caused immediate blockade of further platelet deposition. Since TLR signaling can activate nuclear factor-kappaB (NF-*κ*B), the IKK-inhibitor (IKK inhibitor VII) and NF-*κ*B inhibitor (Bay 11-7082) were tested. The IKK/NF-*κ*B inhibitors strongly inhibited platelet deposition under flow. Furthermore, addition of Pam3CSK4 (TLR1/2 ligand), MALP-2 (TLR2/6 ligand), and Imquimod (TLR7 ligand) reduced phosphotidylserine (PS) exposure. Activation of TLR1/2, TLR2/6, TLR3, TLR7, and TLR9 in whole blood reduced platelet deposition under flow on collagen; however, LPS (major Gram negative bacterial pathogenic component) activation of LTR4 was clearly prothrombotic.

## 1. Introduction

Platelets are not only involved in hemostasis but may also contribute to systemic inflammatory processes that lead to tissue injury [1, 2]. Among characteristics of platelets as immune participating cells, the expression of toll-like receptors (TLRs) is of particular importance. In general, TLR signaling participates in the innate immune system with their function of transmitting danger signals and mediating inflammatory events so as to recruit and activate cells of the adaptive immune system in response to invading pathogens [3]. TLRs have been implicated in the pathogenesis of sepsis [4], atherosclerosis [5, 6], thrombosis [7], hemorrhagic shock [8], trauma [9], periodontitis [10, 11], thrombocytopenia associated with viral infection [12, 13], and acute coronary syndrome [14].

In nucleated cells, TLRs (except TLR3) are mediated by myeloid differentiation primary response gene 88 (MyD88) adaptor protein and the downstream signaling pathway include serine-threonine kinases, interleukin-1 receptor-associated kinase (IRAK), and the tumor necrosis factor (TNF) R-associated factor 6 (TRAF6), the nuclear factor-kappaB (NF-*κ*B) and protein kinases (MAPK), which promotes the expression of various proinflammatory cytokines (e.g. interleukin-6 (IL-6), IL-8, and TNF-alpha) [15, 16]. However, platelets lack a nucleus cell and lack gene regulation, therefore, how TLRs in platelets might be involved in inflammation is less clear.

All 10 TLRs transcripts have been found in platelets and it is reported that the level of some platelet TLRs transcripts has a positive relationship with coronary heart disease risk factors, such as obesity, gender, and inflammation [3, 5]. Not all TLRs are involved in platelet function; only TLR1, 2, 3, 4, 6, 7, and 9 have been reported to be involved in platelet function [3], so only those TLR pathways were tested in this paper.

**Figure 1:**
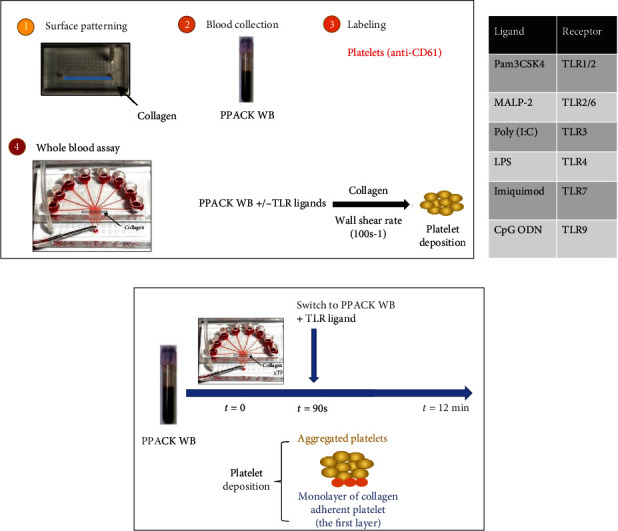
Microfluidics assay, TLR ligands, and switching experiment. Collagen surfaces were patterned on glass slides using single channel patterning devices, then PPACK WB was collected from healthy donors, platelets were labeled with anti-CD61, and actual flow assay were carried out in 8-channel devices (a). TLR ligands and their corresponding TLRs are listed in a table (b). For switching experiment, setting up procedures are same as the flow assay, experiments were running with control condition WB for the first 90s, then switch to the WB with TLR ligands. Monolayer of collagen adherent platelet was formed for the first 90s, then platelets from WB with TLR ligands will aggregate on the first platelet layer.

While a number of studies have investigated the function of TLRs in platelets, the results are somewhat inconsistent. Ward et al. showed that Pam_3_CSK_4_ (TLR1/2 ligand) and LPS (TLR4 ligand) cannot induce calcium mobilization [17], while Fung et al. saw calcium mobilization with Pam_3_CSK_4_ stimulation [18]. Blair et al. concluded that Pam_3_CSK_4_ can induce platelet aggregation [19] and Parra-Izquierdo et al. showed that TLR2 ligand Pam_2_CSK_4_ promotes platelet-endothelial cell interactions [20]; however, Abhilasha et al. showed that neither Pam_3_CSK_4_ nor LPS led to aggregation of platelets. Furthermore, when either Pam_3_CSK_4_ or LPS was added with platelet activating factor, the aggregation percentage decreased [21]; Rivadeneyra et al. found that IKK inhibitors can inhibit platelet aggregation [22], while Gambaryan et al. found that IKK inhibitors can potentiate thrombin- and collagen-stimulated platelet aggregation [23]. Thus, we aimed to investigate the role of TLRs in the context of human whole blood (WB) ex vivo under venous flow conditions since most prior studies relied on platelet rich plasma rather than whole blood. Research done with the in vitro microfluidic test has revealed that a perfusion-switch design (Figure 1) can be employed to study the characteristics of clots at various stages of deposition.

## 2. Methods

### 2.1. Materials

Reagents were obtained as follows: anti-human CD61 antibody (BD Biosciences, San Jose, CA. Cat#: 555754), Alexa Fluor 647–conjugated human fibrinogen (Life Technologies, Grand Island, NY. Cat#: F35200), Alexa Fluor 488-conjugated annexin V (ThermoFisher Scientific, Waltham, MA. Cat#: A13201), collagen (type I; Chrono-Log, Havertown, PA. Cat#: 385), Dade Innovin lipidated tissue factor (TF, Siemens, Malvern, PA, USA), Sigmacote® (Millipore Sigma, Burlington, MA. Cat#: SL2-100ML), Phe-Pro-Arg-chloromethylketone (PPACK, Haematologic Technologies, Essex Junction, VT. Cat#: FPRCK-01), corn trypsin inhibitor (CTI, Haematologic Technologies, Essex Junction, VT, USA), Pam_3_CKS_4_ (NOVUS Biologicals, CO. Cat#: NBP2-25297), MALP-2 (NOVUS Biologicals, CO. Cat#: NBP2-26219), Polyinosinic-polycytidylic acid HMW (NOVUS Biologicals, CO. Cat#: NBP2-25288), LPS (NOVUS Biologicals, CO. Cat#: NBP2-25295), Imiquimod (NOVUS Biologicals, CO. Cat#: NBP2-26228), CpG oligodeoxynucleotides (NOVUS Biologicals, CO. Cat#: NBP2-26232), Vesatolimod (MedChemExpress, NJ. Cat#: HY-15601), GSK2245035 (MedChemExpress, NJ. Cat#: HY-118250), Bay 11-7082 (Millipore Sigma, MO. Cat#: B5556), and IKK inhibitor VII (Millipore Sigma, MO. Cat#: 401486).

### 2.2. Preparation and Characterization of Collagen/TF Surface

Glass slides were first washed with ethanol and then with deionized water before being dried with filtered air. Sigmacote® was then used to create a hydrophobic surface on the glass. 5 *μ*L of fibrillar collagen was run through a microfluidic device to form a 250 *μ*m wide and 60 *μ*m high patterning channel. For experiments without thrombin interaction, the collagen was rinsed and blocked with 20 *μ*L 0.5% bovine serum albumin (BSA) buffer. In experiments that study the effect of thrombin, lipidated TF was adsorbed to the collagen surface by perfusing 5 *μ*L of Dade Innovin PT reagent (20 nM stock concentration), then rinsed and blocked with 20 *μ*L 0.5% BSA, followed by a 30-minute incubation without flow [24, 25].

### 2.3. Blood Collection and Preparation

Blood was collected from healthy donors who had not consumed alcohol in the last 72 hours and had not taken any medications in the past week through a syringe containing PPACK (100 *μ*mol/L) or high concentration of CTI (40 *μ*g/mL). All donors gave their consent with the approval from the University of Pennsylvania Institutional Review Board. After blood collection, CD61 antibody with fluorescent properties was added for platelet labeling. White blood cells are not present in the platelet deposits on collagen under flow until a much later time after the channel is blocked. Even at a low resolution, white blood cell staining would be easily visible if it were there. Experiments with microfluidic perfusion assay were started within 5 minutes of the blood being taken.

### 2.4. Microfluidic Clotting Assay on Collagen Surfaces

A vacuum-sealed 8-channel PDMS flow device was placed perpendicular to collagen surfaces, creating 8 prothrombotic patches (250 x 250 *μ*m). The height of the channel was 120 *μ*m, and the wall shear rate was set to simulate venous conditions (100 s^−1^) [24, 25]. Blood that had been treated with a TLR agonist was added to the inlet reservoir and quickly changed the perfusion pharmacology in about 15 seconds, without any crosstalk between the channels. The clotting events were initiated simultaneously in the microfluidic device, with the initial wall shear rate being regulated by a syringe pump. Platelet activity was monitored by epifluorescence microscopy (IX81) at 10X magnification. Blood from at least three donors was used for each set of experiments, and the total platelet clot mass was determined by fluorescence intensity. Bright field imaging was not used since the thickness of the flowing blood above the clot changed with time. Images were captured using a charged coupled device camera (Hamamatsu, Bridgewater, NJ) and analyzed using ImageJ software. To avoid side-wall effects, only the central 75% of the channel was taken into consideration.

### 2.5. Switching Experiment

The preparation process for the switching experiment was identical to what was described in the prior studies [24, 25]. In the control condition, whole blood was perfused for the initial 90 seconds, and then the TLR ligands were added to the inlet reservoir without ceasing flow. This enabled a fast alteration of perfusion pharmacology within 15 seconds, while also preventing hemodynamic interference between the channels during the perfusion switch. This allowed the first layer of collagen adherent platelets to form within the first 90 seconds, while additional platelets formed in contact with collagen beyond the first layer (Figure 1(c)).

## 3. Results

### 3.1. TLR Ligands (except LPS) Attenuate Platelet Deposition onto Collagen under Flow

**Figure 2:**
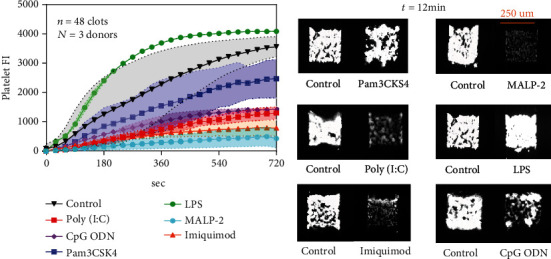
TLR ligands (except LPS) inhibit platelet deposition onto collagen under flow. PPACK WB treated with HBS (control) or TLR ligands was perfused over collagen at 100 s^−1^ for 720 seconds. CD61 was added to all channels to label platelets. Platelet FI was measured throughout the course of the experiments (a) and Figure 2(b) shows how they looked under the microscope at the end point.

To mimic venous hemodynamic conditions in the human body, an eight-channel microfluidic device was used to run control and agonist treated samples simultaneously. PPACK-treated whole blood (no thrombin activity) was perfused over collagen at 100 s^−1^ initial wall shear rate. This assay condition produces strong platelet Glycoprotein VI (GPVI) signaling driven by collagen on the glass substrate [24, 25], especially during the first 90 sec when the first layer of platelets is depositing. WB with HBS was used as control condition and it was observed that most TLR agonists reduced the platelet fluorescent intensity (FI) compared to the matched HBS-WB control condition (Figure 2). MALP-2 was especially potent over the entire time course of the experiment, indicating that TRL-2/6 activation of platelets reduced both primary and secondary deposition. MALP-2 stimulation of whole blood resulted in only a sparse monolayer of platelets by 12 min (Figure 2(b)). Clot buildup was also strongly attenuated by addition of imiquimod, poly (I:C), and CpG ODN, with Pam3CSK4 being mildly inhibitory. While these agonists may also target other responsive cells in whole blood, the final readout is platelet-specific and platelet function is clearly attenuated in the assay. Only LPS promoted platelet deposition under flow (Figure 2(a)).

**Figure 3:**
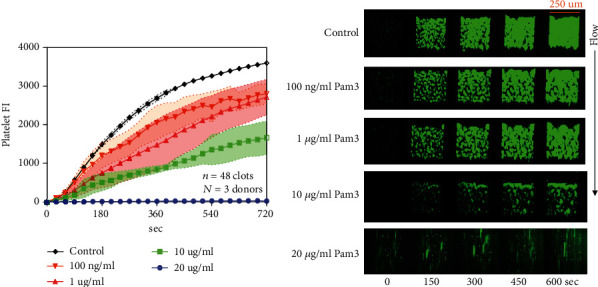
Pam3CSK4 dose dependently inhibit platelet deposition onto collagen under flow. PPACK WB treated with HBS (control) or different concentrations of Pam3CSK4 was perfused over collagen at 100 s^−1^ for 720 seconds. CD61 was added to all channels to label for platelets. Platelet FI was measured throughout the course of the experiments (a) and Figure 3(b) shows how they looked under the microscope.

To investigate whether the concentration of Pam_3_CSK_4_ (TLR1/2 agonist) can produce a dose-dependent inhibition of platelet deposition; different concentrations of Pam_3_CSK_4_ were tested using the microfluidic device. As the concentration of Pam3CSK4 was increased, the platelet FI decreased (Figure 3(a)), with essentially complete blockade of platelet deposition at 20 g/mL Pam3CSK4.

### 3.2. Activation of TLR7 Can Strongly Inhibit Platelet-Platelet Aggregation

It is observed that TLR ligands reduced platelet deposition, to further investigate if this only effect on initial platelet monolayer or also platelet-platelet aggregation, we conducted a series of experiments with blood switching at 90 s using the microfluidic device. The initial procedure was same as before: WB was used to run the experiment at the first 90 s, then, WB samples with TLR ligands were put into the inlet reservoirs without pausing the flow, which enabled a fast change in perfusion pharmacology within 15 seconds and prevented any hemodynamic crosstalk between channels during the perfusion switch. In this way, a monolayer of collagen adherent platelet formed within the first 90 s (the first layer) and then aggregated platelets that generated from TLR ligands WB formed on the first layer (Figure 1(c)).

**Figure 4:**
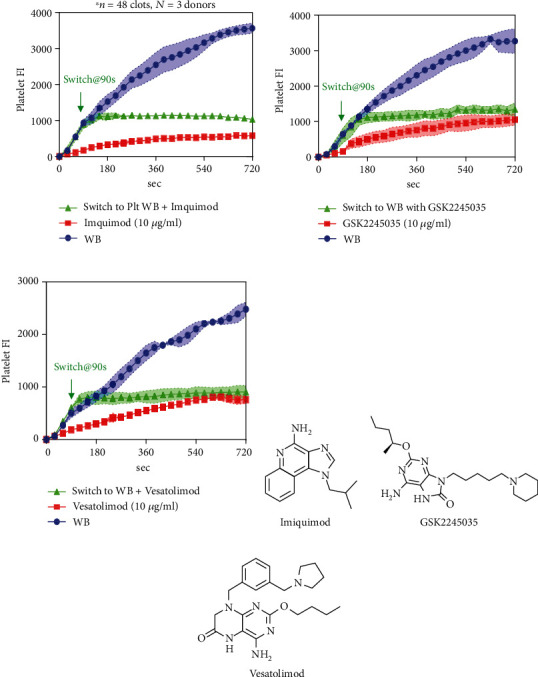
Activation of TLR7 can strongly inhibit platelet-platelet aggregation. PPACK WB treated with control (HBS); TLR7 ligands were perfused over collagen at 100 s^−1^ with or without switching. CD61 was used to label platelets. Fluorescence intensities for platelets were measured throughout the course of the experiments for Imquimod (a), GSK2245035 (b), and Vesatolimod (c). Molecular structure of different TLR 7 ligands were shown in Figures 4(d), 4(e), and 4(f).

Most TLR ligands that we studied can inhibit platelet-platelet aggregation, and the inhibition effect is similar to that of platelet deposition on collagen. While for Imquimod (TLR7 ligand), once we switched blood, the platelet FI never increased, it can strongly inhibit platelet-platelet aggregation (Figure 4(a)). To further investigate if only Imquimod can strongly inhibit platelet-platelet aggregation or it happens on other TLR7 ligands as well, two other different kinds of TLR7 ligands (GSK2245035 and Vesatolimod) were used to run the switching experiment. Similar results were observed as Imquimod: once we switched WB to WB with TLR7 ligand, the platelet FI stopped increasing (Figures 4(b) and 4(c)). Hence, conclusion can be drawn that activation of TLR7 can strongly inhibit platelet-platelet aggregation. The kinetics of inhibition was extremely rapid, demonstrating a role for TLR7 signaling to attenuate platelet-platelet interactions well after the first monolayer of collagen-adherent platelets was formed.

### 3.3. NF-*κ*B/IKK Inhibitors Can Strongly Inhibit Platelet Deposition under Flow

**Figure 5:**
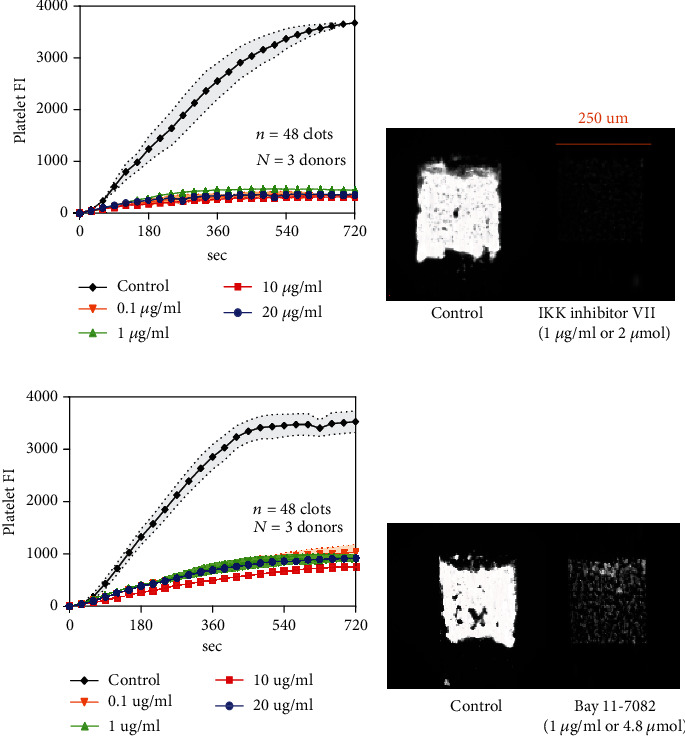
IKK/NF-*κ*B inhibitors can strongly inhibit platelet deposition under flow. PPACK WB with HBS (control) or IKK/NF-*κ*B inhibitors were perfused over collagen at 100 s^−1^ for 720 s. CD61 was added to label platelets. Platelet FI different concentrations of IKK inhibitor VII (a, b) and Bay11-7082 (c, d) were measured throughout the course of the experiments.

From the literature [3], TLR signaling pathways are well recognized to activate the transcription factor NF-*κ*B. Interestingly, we found that most TLR ligands result in the reduction of platelet deposition on collagen. To study the role of NF-*κ*B activation upon TLR activation, NF-*κ*B (Bay11-7082) and IKK inhibitors (IKK inhibitor VII) were used. Microfluidic device was used to test platelet deposition under flow. Different concentrations of different NF-*κ*B and IKK inhibitors were tested, no matter how much of the inhibitors we added into WB, the platelet FI was much lower than the control condition and under the microscope, we saw that only a thin monolayer of platelets was formed (Figures 5(a)–5(d)). Hence, we concluded that NF-*κ*B and IKK inhibitors can strongly inhibit platelet deposition under flow.

### 3.4. Addition of Pam3CSK4, MALP-2, and Imquimod Would Reduce PS Exposure under Flow

**Figure 6:**
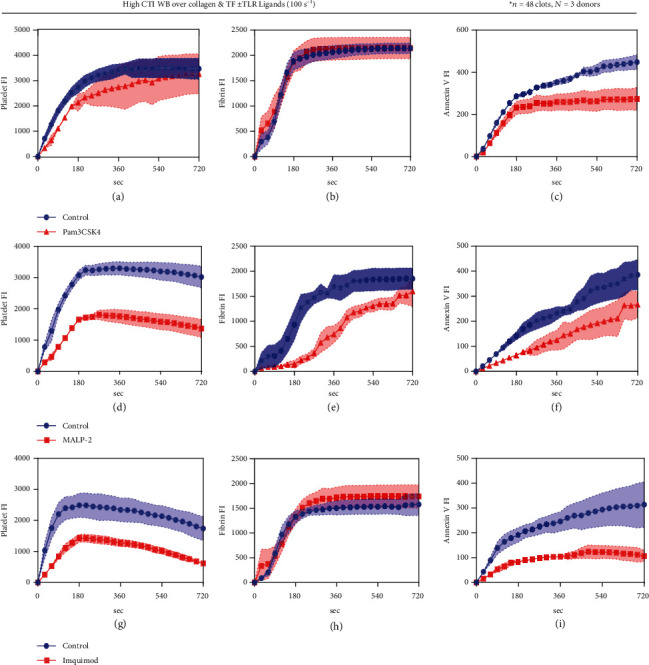
Pam3CSK4, MALP-2, and Imquimod can inhibit PS exposure under flow. High CTI WB with HBS (control) or different TLR ligands was perfused over collagen at 100 s^−1^ for 720 s. CD61, fluorescence fibrinogen fluorophores, and Annexin V fluorophores were added to label for platelets, fibrin, and PS exposure, respectively. Platelet FI (a, d, and g), Fibrin FI (b, e, and h), and Annexin V FI (c, f, and i) were measured throughout the course of the experiments.

The effects of activation TLR in whole blood shown above were obtained with anticoagulated whole blood. We tested TLR agonists in whole blood in a clotting assay that generates thrombin and fibrin via the extrinsic coagulation pathway. Corn trypsin inhibitor (CTI) was used as the anticoagulant to block factor XIIa activity (contact coagulation pathway). We perfused annexin-V and CTI-treated WB ± TLR ligands over collagen/TF (100 s^−1^) and imaged platelet, fibrin, and phosphatidylserine (PS) fluorescence. We observed that addition of Pam3CSK4 (TLR1/2 ligand), MALP-2 (TLR2/6 ligand), and Imquimod (TLR7 ligand) inhibited platelet deposition under flow (Figures 6(a), 6(d), and 6(g)). As for fibrin polymerization, only MALP-2 reduced fibrin FI compared to the control condition (Figures 6(b), 6(e), and 6(h)). Compared to control, we observed less PS exposure with the addition of Pam3CSK4, MALP-2, and Imquimod into WB (Figures 6(c), 6(f), and 6(i)).

## 4. Discussion

A series of experiments were performed with different TLR ligands to investigate the role TLR in hemostasis. Special emphasis was placed on the whole blood context, a minimal surface representative of damaged vessel wall, and the control of hemodynamics using a microfluidic device. Unique to clots forming under flow, the local platelet density in a clot is much greater (>50-fold) than that found in plasma. We found that treatment of whole blood with TLR ligands (expect LPS) reduced platelet deposition under flow, while LPS promoted platelet deposition. In addition, as the concentration of Pam_3_CSK_4_ was increased, the inhibition effect was stronger, a clear dose-dependent response. We observed that most TLR ligands reduced platelet deposition of the initial platelet monolayer. To confirm that this inhibition effect also happened on platelet-platelet aggregation, switching experiments were performed; most TLR ligands also inhibited platelet-platelet aggregation. Activation of TLR7 can strongly inhibit platelet-platelet aggregation.

TLR signaling converges to NF-*κ*B as a result of IKK phosphorylation [1–3]. To study if NF-*κ*B activation has a role in platelet function, NF-*κ*B and IKK inhibitors were tested. It was observed that NF-*κ*B and IKK inhibitors can strongly inhibit platelet deposition under flow. The present study shows that only TLR4 ligand (LPS) has similar as NF-*κ*B in promoting platelet deposition on collagen, while all other TLRs ligands has opposite effects of NF-*κ*B. Defining the molecular signaling linking LPS and NF-*κ*B dependent pathways in platelet is particularly challenging using human whole blood. In Supplemental Figure S4, we used IKK inhibitor-VII and Bay 11-7082 in combination with LPS stimulation of whole blood. Both inhibitors reduce the action of LPS on platelet deposition. Presently, there is not a full understanding of how NF-*κ*B activation leads to changes in platelet function. Studies have demonstrated NF-*κ*B activation will drive platelet activation and that inhibition of NF-*κ*B attenuates platelet activation [22]. The nongenomic functions of NF-*κ*B in platelets are highly unexpected and interesting. Clearly, transcription factor activity has no role in our measurements of platelet function on collagen. Given the short incubation time of 5 min in our experiments, even nucleated white blood cells in the whole blood would not likely have altered gene expression to influence the experiment. As noted earlier, prior studies have clearly demonstrated a nongenomic function of NF-*κ*B in platelets. However, not molecular mechanism has yet been discovered for NF-*κ*B function in platelets. One hypothesis is that inhibitory mechanisms involving cAMP and cGMP may somehow become engaged following platelet activation by TLR agonists, perhaps as a negative feedback mechanism. For example, Gambaryan has studied the role of platelet released nitric oxide (NO) in platelet function [26]. A separate hypothesis is that TLR activation may cause epinephrine release in whole blood to promote platelet exhaustion and reduced deposition on collagen [27]. In future work we will explore these possibilities.

Experiments were conducted to evaluate the influence of TLR activation on the blood clotting process. Annexin V and CTI-treated WB either with or without TLR ligands were pumped over collagen/TF for this purpose. Addition of some TLR ligands (Pam3CSK4, MALP-2, and Imquimod) inhibited platelet deposition under flow, only MALP-2 reduced fibrin FI compared to the control condition and less PS exposure were observed with the addition of some TLR ligands (Pam3CSK4, MALP-2, and Imquimod) into WB. Also, it was noticed that in the experiment with PPACK WB perfused over collagen, LPS promoted platelet deposition under flow; but in the experiment with CTI-treated WB perfused over collagen/TF, LPS weakly inhibited platelet deposition ((available here) Supplement Figures). This is due to the present of thrombin, in the clotting event with LPS, damaged tissue encountered body meditating infection, although the defense mechanism is unknown, resulting in full clotting by thrombin.

As reviewed by Hally et al. [28], the effect of TLR activators on platelets can produce contrasting results depending on the specifics of timing, dose, and composition of the TLR agonist. Platelet TLR levels and signaling are clearly implicated in vascular and infectious disease, but these modulatory roles may present differently at early and late times. Our research demonstrates that TLR activation can have strong, typically attenuating effects on platelets under microfluidic whole blood clotting assay. This would be consistent to a role for TLRs in controlling and regulating the thromboinflammatory setting to prevent exaggerated tissue damaging responses.

While clinicians have many approved antithrombotic drugs to apply toward clinical situations, the targeting of TLRs or TLR downstream signaling in platelets is less developed. We are not aware of any approved TLR antagonist or agonist drugs. In sepsis, trials are in progress to test TLR4 antagonists (Clinical Trial # NCT00334828). Our data indicates that LPS is different from the other agonists in that LPS promotes clotting under flow, suggesting that a TLR4 antagonist could have antithrombotic activity.

In the future, in vivo work using the laser injury model of cremaster arterioles [29], the mouse could be pretreated with LPS prior to laser induced bleeding and hemostasis. In such experiments, the timing between LPS stimulation and the laser injury would be a crucial variable to control, given the potential for platelet exhaustion by LPS stimulation.

## Data Availability

All data used to support the findings of this study are included within the article and supplementary information files.

## Supplementary Materials

Supplementary Materials Supplemental Figure S1: calcium mobilization potential via TLR. Platelet rich plasma (PRP) with HBS was used as negative control and PRP with 10 nM of convulxin was used as positive control. Calcium mobilization of different concentrations of TLR ligands were tested using Fluoroskan (A) and Envision (B). Supplemental Figure S2: platelet aggregation via TLR activation. Platelet aggregometry was used to investigate the role of TLR in platelet aggregation. Collagen at 2 *μ*g/mL was used as positive control, TLR ligands were added into PRP to test platelet aggregation via TLR activation (A). TLR ligands with collagen together were added to PRP to test platelet aggregation (B). Supplemental Figure S3: activation of most TLR ligands can inhibit platelet-platelet aggregation. PPACK WB treated with control (HBS), TLR ligands was perfused over collagen at 100 s-1 with or without switching. CD61 was used to label platelets. Fluorescence intensities for platelets were measured throughout the course of the experiments for Pam3CSK4 (A), MALP-2 (B), Poly (I:C) (C), LPS (D), and CpG ODN (E). Supplemental Figure S4: IKK/NF-KB inhibitors and LPS have opposite effect on platelet deposition. PPACK WB treated with HBS (control) or LPS or IKK inhibitor VII (A) /Bay11-7082 (B) or IKK inhibitor VII/Bay11-7082 with LPS was perfused over collagen at 100 s-1 for 720 seconds. CD61 was added to all channels to label for platelets. Platelet FI was measured throughout the course of the experiment. Figure C showed the hypothesis for the signaling pathway. Supplemental Figure S5: Poly (I:C), LP, and CpG ODN do not inhibit Annexin V binding to PS sites under flow. High CTI WB with HBS (control) or different TLR ligands were perfused over collagen at 100 s-1 for 720 s. CD61, fluorescence fibrinogen fluorophores, and Annexin V fluorophores were added to label for platelets, fibrin, and PS exposure, respectively. Platelet FI (A, D, and G), Fibrin FI (B, E, and H), and Annexin V FI (C, F, and I) were measured throughout the course of the experiments.
