# Supplementary material for: Activation of Most Toll-Like Receptors in Whole Human Blood Attenuates Platelet Deposition on Collagen under Flow
Source: J Immunol Res. 2023 Jan 17;2023:1884439. doi: 10.1155/2023/1884439 (PMC9873445; doi:10.1155/2023/1884439)
Supplement: Supplementary Materials — Supplemental Figure S1: calcium mobilization potential via TLR. Platelet rich plasma (PRP) with HBS was used as negative control and PRP with 10 nM of convulxin was used as positive control. Calcium mobilization of different concentrations of TLR ligands were tested using Fluoroskan (A) and Envision (B). Supplemental Figure S2: platelet aggregation via TLR activation. Platelet aggregometry was used to investigate the role of TLR in platelet aggregation. Collagen at 2 μg/mL was used as positive control, TLR ligands were added into PRP to test platelet aggregation via TLR activation (A). TLR ligands with collagen together were added to PRP to test platelet aggregation (B). Supplemental Figure S3: activation of most TLR ligands can inhibit platelet-platelet aggregation. PPACK WB treated with control (HBS), TLR ligands was perfused over collagen at 100 s-1 with or without switching. CD61 was used to label platelets. Fluorescence intensities for platelets were measured throughout the course of the experiments for Pam3CSK4 (A), MALP-2 (B), Poly (I:C) (C), LPS (D), and CpG ODN (E). Supplemental Figure S4: IKK/NF-KB inhibitors and LPS have opposite effect on platelet deposition. PPACK WB treated with HBS (control) or LPS or IKK inhibitor VII (A) /Bay11-7082 (B) or IKK inhibitor VII/Bay11-7082 with LPS was perfused over collagen at 100 s-1 for 720 seconds. CD61 was added to all channels to label for platelets. Platelet FI was measured throughout the course of the experiment. Figure C showed the hypothesis for the signaling pathway. Supplemental Figure S5: Poly (I:C), LP, and CpG ODN do not inhibit Annexin V binding to PS sites under flow. High CTI WB with HBS (control) or different TLR ligands were perfused over collagen at 100 s-1 for 720 s. CD61, fluorescence fibrinogen fluorophores, and Annexin V fluorophores were added to label for platelets, fibrin, and PS exposure, respectively. Platelet FI (A, D, and G), Fibrin FI (B, E, and H), and Annexin V FI (C, F, and I) were mea [file 1884439.f1.docx]

**Supplement**

**Activation of toll like receptors in whole human blood attenuates platelet deposition on collagen under flow.**

**Y. Liu, S. L. Diamond***

1 Department of Chemical and Biomolecular Engineering

Institute for Medicine and Engineering

University of Pennsylvania

Philadelphia, PA 19104 USA

*Corresponding Author:

Scott L. Diamond, PhD

Department of Chemical and Biomolecular Engineering

Institute for Medicine and Engineering

1024 Vagelos Research Laboratory

University of Pennsylvania

Philadelphia, PA 19104, USA.

Tel: 215-573-5702

fax: 215-573-7227

email: sld@seas.upenn.edu

**Supplemental Method**

**Materials**

Reagents were obtained as follows: anti-human CD61 antibody (BD Biosciences, San Jose, CA. Cat#: 555754), Alexa Fluor 647–conjugated human fibrinogen (Life Technologies, Grand Island, NY. Cat#: F35200), Alexa Fluor 488-conjugated annexin V (ThermoFisher Scientific, Waltham, MA. Cat#: A13201), collagen (type I; Chrono-Log, Havertown, PA. Cat#: 385), Dade Innovin lipidated tissue factor (TF, Siemens, Malvern, PA, USA), Sigmacote® (Millipore Sigma, Burlington, MA. Cat#: SL2-100ML), Fluo-4 NW calcium dye and probenecid (Invitrogen, Carlsbad, CA, USA), Convulxin (CVX, Santa Cruz Biotechnology, Cat# sc-202554), Phe-Pro-Arg-chloromethylketone (PPACK, Haematologic Technologies, Essex Junction, VT. Cat#: FPRCK-01), corn trypsin inhibitor (CTI, Haematologic Technologies, Essex Junction, VT. Cat#: CTI-01), Pam_3_CKS_4_ (NOVUS Biologicals, CO. Cat#: NBP2-25297), MALP-2 (NOVUS Biologicals, CO. Cat#: NBP2-26219), Polyinosinic-polycytidylic acid HMW (NOVUS Biologicals, CO. Cat#: NBP2-25288), LPS (NOVUS Biologicals, CO. Cat#: NBP2-25295), Imiquimod (NOVUS Biologicals, CO. Cat#: NBP2-26228), CpG oligodeoxynucleotides (NOVUS Biologicals, CO. Cat#: NBP2-26232), Vesatolimod (MedChemExpress, NJ. Cat#: HY-15601), GSK2245035 (MedChemExpress, NJ. Cat#: HY-118250), Bay 11-7082 (Millipore Sigma, MO. Cat#: B5556) and IKK inhibitor VII (Millipore Sigma, MO. Cat#: 401486).

**Intracellular Calcium Mobilization**

To investigate calcium mobilization with TLR ligands, platelet-rich plasma (PRP) was isolated from PPACK- treated whole blood via moderate centrifugation (120 g, 10 min, 20^o^C), diluted in HEPES-buffered saline and incubated with Fluo-4 NW fluorescent dye for 30 minutes. One 384-well plate were prepared with dye-loaded PRP and TLR ligands or other reagents were added right before the experiment. Dynamic data was collected from Fluoroskan and Evision.

**Platelet Aggregometry**

Platelet aggregation experiments were performed using a Model 700 Whole Blood/Optical Lumi-Aggregation System (Chrono-log). Healthy PRP or platelets rich plasma (PPP) were prepared from citrated whole blood. TLRs were manually dispensed into sample cuvettes at 37^o^C, and aggregation was monitored for 4 min.

**Microfluidic assay**

For coating with collagen, a single 1000-lm-wide channel (PDMS) patterning device was vacuum-sealed to Sigmacote® -treated glass slide, as previously described [1,2]. Then, 5 µL of 0.5 mg mL-1 collagen solution was perfused through the channel to create a prothrombotic coating on the glass. The patterning device was replaced with an eight-channel PDMS device, with each channel (height, 120 µm; width, 250 µm) positioned perpendicular to the patterned collagen (Fig. 1). PPACK treated blood was perfused across the 8 channels by withdrawal through a single outlet and the shear rate was kept at venous level (100s^-1^). All clotting events were initiated simultaneously in the microfluidic device on the collagen strip. Initial wall shear rate was controlled by a syringe pump (Harvard PHD ULTRA / Harvard PHD 2000; Harvard Apparatus, Holliston, MA) connected to the outlet on the flow device. Platelet were monitored simultaneously by epifluorescence microscopy (IX81; Olympus America Inc., Center Valley, PA) at 10X magnification. For each set of experiments, blood samples from N≥3 donors were taken. Images were captured with a charged coupled device camera (Hamamatsu, Bridgewater, NJ) and were analyzed with ImageJ software (National Institutes of Health). To avoid side-wall effects, fluorescence values were taken only from the central 75% of the channel.

**References**

1 Zhu S, Chen J, Diamond SL. Establishing the Transient Mass Balance of ThrombosisHighlights. Arterioscler Thromb Vasc Biol. 2018;38:1528–36.

2. Alshehri OM, Hughes CE, Montague S, Watson SK, Frampton J, Bender M, et al. Fibrin activates GPVI in human and mouse platelets. Blood. 2015;126:1601–8.

**Supplemental Figures S1-S5**

Supplemental Figure 1.

**A**

**B**

**Supplemental Figure S1. Calcium mobilization potential via TLR.** Platelet Rich Plasma (PRP) with HBS was used as negative control and PRP with 10nM of convulxin was used as positive control. Calcium mobilization of different concentrations of TLR ligands were tested using Fluoroskan (A) and Envision (B).

Supplemental Figure 2


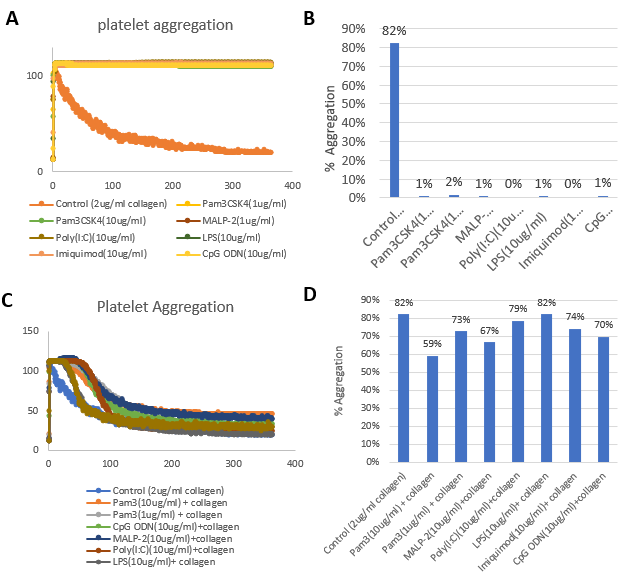


**Supplemental Figure S2.** Platelet aggregation via TLR activation. Platelet aggregometry was used to investigate the role of TLR in platelet aggregation. Collagen at 2 μg/mL was used as positive control, TLR ligands were added into PRP to test platelet aggregation via TLR activation(A). TLR ligands with collagen together were added to PRP to test platelet aggregation (B).

Supplemental Figure 3


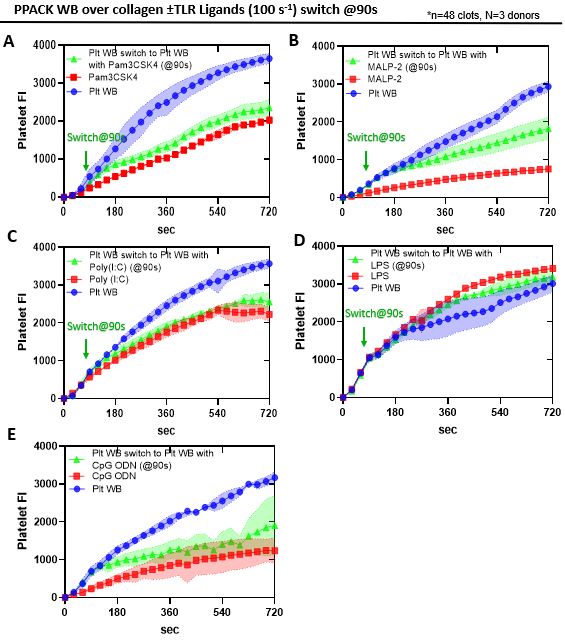


**Supplemental Figure S3. Activation of most TLR ligands can inhibit platelet-platelet aggregation.** PPACK WB treated with control (HBS), TLR ligands was perfused over collagen at 100s-1 with or without switching. CD61 was used to label platelets. Fluorescence intensities for platelets were measured throughout the course of the experiments for Pam3CSK4 (A), MALP-2(B), Poly(I:C) (C), LPS (D) and CpG ODN (E).

Supplemental Figure 4


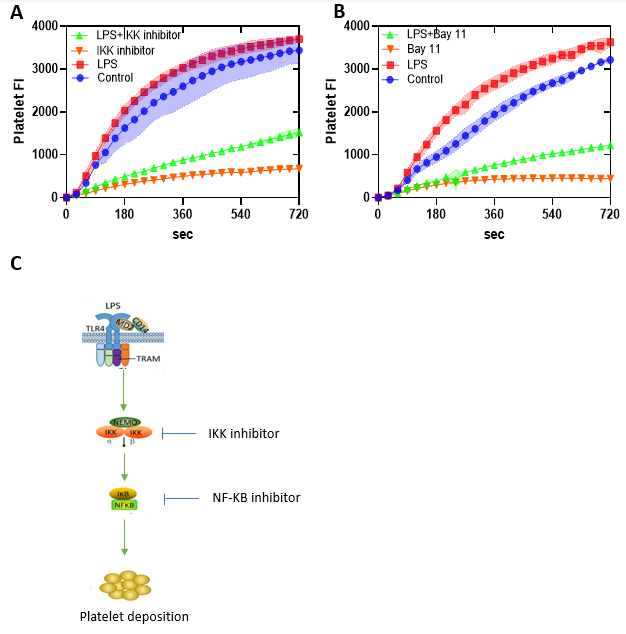


**Supplemental Figure S4. IKK/NF-KB inhibitors and LPS has opposite effect on platelet deposition.** PPACK WB treated with HBS (control) or LPS or IKK inhibitor VII (A)/Bay11-7082(B) or IKK inhibitor VII/Bay11-7082 with LPS was perfused over collagen at 100s-1 for 720 seconds. CD61 was added to all channels to label for platelets. Platelet FI were measured throughout the course of the experiment. Figure C showed the hypothesis for the signaling pathway.

Supplemental Figure 5


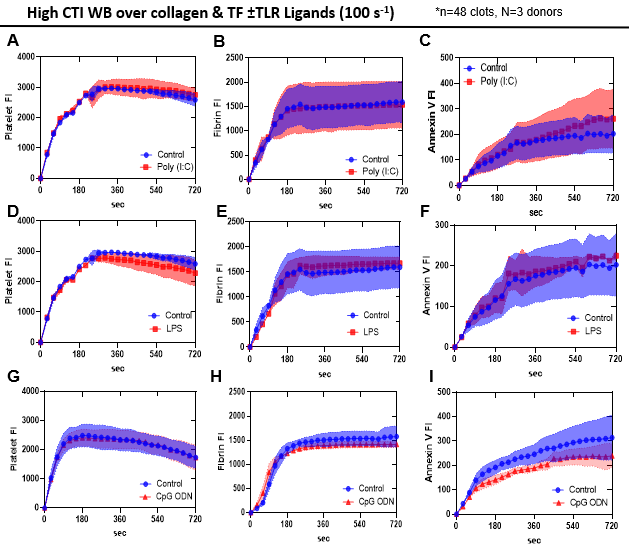


**Supplemental Figure S5. Poly(I:C), LP and CpG ODN do not inhibit Annexin V binding to PS sites under flow.** High CTI WB with HBS (control) or different TLR ligands was perfused over collagen at 100s-1 for 720s. CD61, fluorescence fibrinogen fluorophores and Annexin V fluorophores were added to label for platelets, fibrin and PS exposure, respectively. Platelet FI (A,D,G), Fibrin FI (B,E,H) and Annexin V FI (C,F,I) were measured throughout the course of the experiments.
